# Importance of integrating epidemiological and genomic surveillance of seasonal influenza viruses to monitor global circulation

**DOI:** 10.1002/ctm2.70126

**Published:** 2024-12-02

**Authors:** Zhiyuan Chen, Hongjie Yu

**Affiliations:** ^1^ School of Public Health Key Laboratory of Public Health Safety Ministry of Education, Fudan University Shanghai China; ^2^ Shanghai Institute of Infectious Disease and Biosecurity Fudan University Shanghai China; ^3^ Department of Infectious Diseases Huashan Hospital, Fudan University Shanghai China

1

The seasonal influenza virus is an acute viral respiratory pathogen that circulates around the globe annually. A modelling study estimated that every year there are 290 000–650 000 seasonal influenza‐associated respiratory deaths,^1^ with the highest risk among the elderly, young children, people with chronic medical conditions, and pregnant women.^2^ Vaccination is the most important prevention strategy, but influenza vaccine should be administered annually due to frequent viral antigenic drift. Each year, the World Health Organization recommends seasonal influenza vaccine strain compositions—for the northern hemisphere in February and the southern hemisphere in September.

Seasonal influenza has a fairly regular circulation pattern, with winter epidemics in temperate regions and diverse seasonality in tropical and subtropical areas.^3^ Previous work hypothesized a source‐sink dynamic model for the ecology of seasonal influenza virus in which annual epidemics in temperate regions are generally seeded from persistent influenza reservoirs.^4^ In 2015, the global circulation patterns of seasonal influenza virus and their potential correlates were further characterized, deepening the understanding of the epidemic spread of seasonal influenza.^5^

Changes in coronavirus disease 2019 (COVID‐19) pandemic‐related human behaviours heavily shaped activity levels of seasonal influenza.^6^ During periods with stringent implementation of non‐pharmaceutical interventions (NPIs), from April 2020 to March 2021, influenza virus test positivity rates decreased globally and B/Yamagata lineages were rarely detected after March 2020.^7^ Following recovery of travel and population mixing and with the exception of B/Yamagata lineages, there was a resurgence of seasonal influenza virus activity, albeit with spatial heterogeneity.

An unresolved question is how global dispersal patterns of seasonal influenza viruses were disrupted during the COVID‐19 pandemic and how they were restored. Our research team recently estimated the underlying influenza landscape by combining global virological surveillance data, genetic sequence data, and air traffic data from 2017 to 2024.^8^ Twelve geographic regions were defined and contextualized into analyses of four periods: the pre‐pandemic period (January 2017–March 2020), the acute phase of the pandemic (April 2020–March 2021), the transition phase of the pandemic (April 2021–April 2023), and the post‐pandemic period (May 2023–March 2024).

Seasonal influenza virus activity and degree of air traffic‐based human movement both experienced “decline and recovery” during the pandemic. Time‐inhomogeneous phylodynamic analyses identified air traffic‐based human mobility as the principal driver of global influenza virus dissemination, despite a major reduction of influenza activity during the pandemic. Analyses of trunk locations along the phylogenetic trees suggested that South Asia and West Asia were more likely to play key roles in maintaining the circulation of influenza A and B/Victoria lineages, respectively, during the pandemic. Importantly, the global dissemination pattern and intensity of seasonal influenza appears to have reverted to pre‐pandemic levels during the post‐pandemic period.

Dwell times (also referred to as persistence times) of seasonal influenza A/H3N2 virus were estimated for Africa, Southeast Asia, and South Asia. Dwell times may be useful for predicting the distribution of circulating strains and informing seasonal influenza vaccine composition.^9^ Increased dwell time was apparent during the acute phase of the pandemic, indicating independent virus evolution and circulation. Further statistical analysis revealed associations with antigenic drift and air traffic‐based human movement. Our research team also conducted analyses of genetic diversity and selection pressures over time to provide insights into the possible “extinction” of B/Yamagata lineages.

We believe that our team's study enhances the understanding of the global spread of seasonal influenza viruses, specifically identifying dissemination patterns and key transmission areas and drivers that were revealed through analyses of their disruption by the COVID‐19 pandemic and NPIs. The invited Perspective insights from professors Pejman Rohani and Justin Bahl emphasize the effectiveness of NPIs in disrupting viral transmission, pathogen diversity, and antigenic evolution of seasonal influenza viruses.^10^ The rapid and robust recovery of global lineage dispersal patterns after the perturbation associated with the COVID‐19 pandemic highlights the importance of strengthening virological and genomic surveillance of respiratory pathogens worldwide. Empirical evidence from these findings could be helpful for comprehensive responses to future pandemics and could deepen the understanding of the transmission and evolution of seasonal respiratory pathogens under various pandemic scenarios and mitigation strategies. These findings may also provide information helpful for influenza treatment and clinical management. The wide variety of seasonal influenza evolution and circulation patterns by subtype/lineage, region, and time necessitate tailored, individual‐based treatment options that could be informed with updated information about the genetic and antigenic characteristics of seasonal influenza viruses.

Further research is needed to couple the mathematical model into analyses that provide mechanistic insights, especially exploring the extent of disruption that specific pandemic‐related NPIs have on seasonal influenza viruses and other respiratory pathogens. Multiscale and multisource modelling frameworks enable accommodating virus evolution, population immunity, and human movement into a holistic analysis of viral spread. These frameworks could explore the specific contributions of each factor and further inform control strategies against annual seasonal influenza epidemics—vaccination, medical intervention, and NPIs implementation.

## CONFLICT OF INTEREST STATEMENT

Hongjie Yu received research funding from Sanofi Pasteur, GlaxoSmithKline, Yichang HEC Changjiang, Shanghai Roche Pharmaceutical Company, and SINOVAC Biotech Ltd. None of these funds are related to this work. Zhiyuan Chen declares no conflict of interest.
